# The Emerging Role of Long Non-Coding RNAs in the Metastasis of Hepatocellular Carcinoma

**DOI:** 10.3390/biom10010066

**Published:** 2019-12-31

**Authors:** Xuejiao Chen, Feng-Ru Tang, Frank Arfuso, Wen-Qi Cai, Zhaowu Ma, Jiyuan Yang, Gautam Sethi

**Affiliations:** 1 The First School of Clinical Medicine, Health Science Center, Yangtze University, Nanhuan Road, Jingzhou 434023, China; chenxuejiaochina@163.com; 2 Radiation Physiology Laboratory, Singapore Nuclear Research and Safety Initiative, National University of Singapore, Singapore 138602, Singapore; snrtfr@nus.edu.sg; 3 Stem Cell and Cancer Biology Laboratory, School of Pharmacy and Biomedical Sciences, Curtin Health Innovation Research Institute, Curtin University, Perth, WA 6009, Australia; frank.arfuso@curtin.edu.au; 4 School of Basic Medicine, Health Science Center, Yangtze University, 1 Nanhuan Road, Jingzhou 434023, China; cwq7537@163.com; 5 Department of Pharmacology, Yong Loo Lin School of Medicine, National University of Singapore, Singapore 117600, Singapore

**Keywords:** hepatocellular carcinoma, long non-coding RNAs, metastasis, epigenetics, transcription

## Abstract

Long non-coding RNAs (lncRNAs) play multifaceted roles in modulating gene expression under both physiological and pathological processes. The dysregulation of lncRNAs has been increasingly linked with many human diseases, including a plethora of cancers. Mounting evidence indicates that lncRNAs are aberrantly expressed in hepatocellular carcinoma (HCC) and can regulate HCC progression, as well as metastasis. In this review, we summarize the recent findings on the expanding roles of lncRNAs in modulating various functions of HCC, and elaborate on how can lncRNAs impact HCC metastasis and progression via interacting with chromatin, RNA, and proteins at the epigenetic, transcriptional, and post-transcriptional levels. This mini-review also highlights the current advances regarding the signaling pathways of lncRNAs in HCC metastasis and sheds light on the possible application of lncRNAs for the prevention and treatment of HCC.

## 1. Introduction

The long non-coding RNAs (lncRNAs) are widely defined as a large class of regulatory transcripts with a length of more than 200 nucleotides and lacking obvious protein-coding potential [1]. A large body of studies have shown that lncRNAs can functions as critical mediators in the basic physiological and pathological processes, as well as cancer metastasis [2,3]. To date, a wealth of independent research has indicated that the dysregulation of lncRNAs exerts a significant role in hepatocellular carcinoma metastasis. However, the potential molecular mechanisms of lncRNAs in HCC metastasis are not comprehensively understood.

Hepatocellular carcinoma (HCC) is the fourth-leading cause of cancer death in the world, with 841,000 new cases and 782,000 deaths each year [4]. Although recent diagnostic methods and surgical techniques has been significantly improved, the five-year survival rate for advanced HCC remains low. The disappointing clinical outcome of HCC is mainly due to the high metastases or recurrence rate [5]. Therefore, in order to promote the research progress of HCC, especially advanced HCC with metastasis, elucidating the possible molecular mechanisms of malignant biological behaviors of tumor cells is particularly important.

Several recent reviews focus on the emerging role of lncRNAs in HCC [6,7,8]. The main aspect of our review is to summarize the increasingly recognized functions of lncRNAs in HCC metastasis. We highlight a broad repertoire of functions and molecular mechanisms of lncRNAs at the epigenetic, transcriptional, and post-transcriptional levels in HCC metastasis. Moreover, we briefly elaborate on the current advances regarding the signaling pathways of lncRNAs in HCC metastasis and the role of lncRNAs in the multi-step process of HCC metastasis.

## 2. LncRNAs as Novel Regulators in HCC Metastasis

LncRNAs are a large class of non-coding RNA transcripts, which are increasingly known to be important for the development and progression of chronic diseases. LncRNAs act as versatile regulators that can interact with RNA, DNA, or proteins to modulate gene expression at different molecular levels, including epigenetic modification, transcriptional, and post-transcriptional regulation [9]. Currently, there are four accepted general archetypes for classifying the functions of lncRNAs: signals, decoys, guides, and scaffold [3,10]. As signals, lncRNAs can regulate gene regulation in a spatiotemporal way via reflecting the combinatorial biological outcome of transcription factors (TFs) or signaling pathways. As decoys, lncRNAs bind and titrate TFs and diverse proteins away from chromatin or titrate the protein into nuclear subdomains. As guides, lncRNAs may recruit RNA-binding proteins (RBPs) to target genes, either in cis or in trans. As flexible scaffolds, lncRNAs can also absorb different macromolecules, form complexes, and play different biological functions. Many recent reports have revealed that lncRNAs are involved in the multiple biological functions of tumor cells, such as proliferation, invasion, metastasis, and drug resistance [11,12,13,14,15].

Mounting evidence indicates that abnormal expression of lncRNAs can impact multiple steps of HCC progression, including HCC metastasis. A well-described example for functionally important lncRNA in HCC metastasis was termed HULC (highly up-regulated in liver cancer), which was related with highly specific up-regulation characterized in HCC tissues and associated with intrahepatic metastases, tumor node metastasis (TNM) stage, and HCC recurrence [16]. Another lncRNA MVIH (microvascular invasion in HCC) has also been proved to be an oncogenic regulator that is responsible for tumor growth and can serve as a key regulatory center in HCC intrahepatic metastasis by activating angiogenesis. Moreover, the overexpressed MVIH can serve as an independent risk factor for predicting poor RFS (recurrence-free survival) after a hepatectomy [17] LncRNAs may function as a novel participant in the complex steps of HCC metastasis (Table 1). Thus, summarizing the current findings of lncRNAs will enhance our understanding related to the role of lncRNAs in metastasis and may assist in formulating lncRNA-related therapeutic strategies against HCC.

## 3. The Molecular Mechanisms of LncRNAs in HCC Metastasis

### 3.1. LncRNAs in HCC Metastasis at the Epigenetic and Transcriptional Level

#### 3.1.1. Chromatin Modification and Regulation

**Figure 1:**
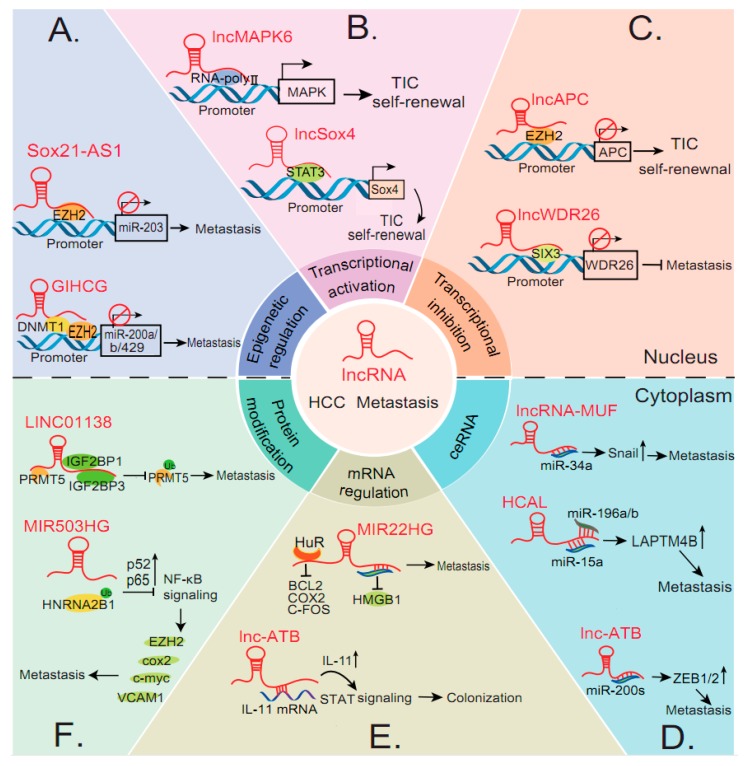
LncRNAs regulate hepatocellular carcinoma metastasis at different molecular levels. LncRNAs play a pivotal role in gene regulation and exert their effects in hepatocellular carcinoma metastasis through diverse mechanisms, including (**A**) epigenetic modification, (**B**,**C**) transcriptional regulation, and (**D**–**F**) post-transcriptional regulation.

Accumulating studies show that lncRNAs may exert a diverse range of functions to regulate gene transcription involved in epigenetic modifiers in HCC [46,47]. Histone methylation and DNA methylation, as important epigenetic modification manners, are involved in modulating the expression of numerous cancer-related genes [48,49]. For instance, a recent study revealed that linc-GALH (Gankyrin associated lincRNA in hepatocellular carcinoma), which might be a new biomarker for judging HCC metastasis, could promote the degradation of DNMT1 (DNA methyltransferase 1) by enhancing the ubiquitination and expression of Gankyrin (standard nomenclature is PSMD10) by lowering the methylation state in HCC [18]. Considerable evidence suggests that the EMT (epithelial–mesenchymal transition) is responsible for the metastasis and recurrence of tumors, including HCC. Moreover, several lncRNAs have recently been proved to be associated with epigenetic regulators to mediate the expression of EMT-related genes or microRNAs. LncRNA GIHCG (gradually increased during hepatocarcinogenesis) physically binds with an EZH2 (Enhancer of Zeste Homolog 2) and miR200b/a/429 promoter, recruits EZH2 and DNMT1 to the miR-200b/a/429 promoter regions, upregulates histone H3K27 trimethylation and DNA methylation levels on the miR200b/a/429 promoter, and significantly inhibits the expression of miR-200b/a/429 expression (Figure 1A) [19]. Thus, GIHCG promotes proliferation and metastasis via inhibiting transcription of miRNA clusters in HCC. Inversely, lncRNA H19 inhibits HCC metastasis and the expression of markers of EMT by associating with the protein complex hnRNP U/PCAF/RNA Pol II and activating the miR-200 family through increasing histone acetylation [27]. Another study has shown that reducing lncRNA SOX21-AS1 (SOX21 antisense divergent transcript 1) expression facilitates HCC metastasis by epigenetically silencing p21 via recruiting EZH2 to the promoter of p21 (Figure 1A) [20]. Together, these studies indicated that increasing lncRNAs can interact directly or indirectly with epigenetic regulator EZH2 to modulate gene expression, thereby affecting the metastasis of HCC.

Liver tumor initiating cells (TICs), a small subset of cells in HCC, are considered to account for the origin, metastasis, and recurrence of HCC [50]. Recent studies have revealed that lncRNAs exert the critical roles in maintaining TIC self-renewal. For example, LncFZD6, which is overexpressed in liver cancer and liver TICs, drives liver TIC self-renewal and tumor initiation capacity. Mechanistically, lncFZD6 directly binds and recruits the BRG1 (Brahma-related Gene 1 protein)-embedded the switch/sucrose non-fermenting (SWI/SNF) complex to the FZD6 promoter, which facilitates the transcription of FZD6 via chromatin remodeling [21]. Similarly, the overexpressed lncHOXA10 recruits the nucleosome remodeling factor (NURF) chromatin remodeling complex to the HOXA10 promoter to initiate the expression of HOXA10, and ultimately promotes the self-renewal of liver TICs and the progression of HCC [22]. Collectively, increasing studies have shown that lncRNAs epigenetically regulate gene transcription by manipulating DNA methylation and histone acetylation. Additionally, lncRNAs modulate the transcription of neighbor or distant genes in cis or trans form by recruiting various remodeling complexes to gene promoters, resulting in activation or inhibition of HCC metastasis-related genes.

#### 3.1.2. Transcriptional Regulation

For transcription regulation, lncRNAs may bind to the promoter of neighbor or distant genes, and recruit transcription factors (TFs) to manipulate transcriptional initiation [3]. LncSox4 is overexpressed in liver cancer and liver TICs, especially in advanced liver tumor. LncSox4 promotes liver TIC self-renewal by driving Sox4 transcription via recruiting Stat3 to the Sox4 promoter (Figure 1B) [23]. Moreover, a novel lncRNA-NEF (neighboring enhancer of FOXA2) can significantly antagonize EMT progression and cancer metastasis. It can be transcriptionally activated by EMT suppressor FOXA2 via interacting with β-catenin and triggering β-catenin inhibitory phosphorylation, thereby attenuating Wnt/β-catenin signaling and activating FOXA2 expression, which may form a positive feedback loop and thus regulating HCC metastasis [26]. Another novel lncRNA called lncRNA-AWPPH, which is highly expressed in HCC tissues, as well as in liver metastatic portal vein tumor thrombus (PVTT) tissues, promotes YBX1-mediated activation of SNAIL1 translation and PIK3CA transcription, and activates the PI3K/AKT pathway. The Pearson chi-square test was used to analyze the correlations between the expression of lncRNA-AWPPH and clinicopathological features in 88 HCC patients. The results showed that a high expression of lncRNA-AWPPH was correlated with microvascular invasion (*p* = 0.033; *p* < 0.05 denotes significance) [35]. Additionally, a divergent lncRNA of Mitogen-activated protein kinase 6 (MAPK6), called lncMAPK6, is highly expressed along with liver tumorigenesis. It interacts with and recruits RNA polymerase II to be a MAPK6 promoter, and finally activates the transcription of MAPK6 (Figure 1B) [21].

On the contrary, lncAPC inhibits transcription of APC via recruiting EZH2 to be a APC promoter, which facilitates the activation of Wnt/β-catenin signaling and liver TIC self-renewal [24] (Figure 1C). Overexpression of lncWDR26 (GenBank Accession no. RP11-365O16) can suppress HCC growth and metastasis through the inhibition of WDR26 transcription via association with SIX3 (Figure 1C) [25]. Taken together, lncRNAs act as versatile molecules to activate or inhibit metastasis-related genes at the transcriptional level.

### 3.2. LncRNAs in HCC Metastasis at the Post-Transcriptional Level

#### 3.2.1. Interactions with miRNAs

Over the past decade, with the continuous development of biotechnology, competing endogenous RNA (ceRNA) has emerged as a common molecular mechanism involving tumor-related lncRNAs. There has been considerable evidence suggesting that several lncRNAs are involved in regulating gene expression via interacting with miRNAs, thus preventing specific miRNAs from binding to their target mRNA [51,52,53]. For instance, lncRNA HCAL (HCC-associated lncRNA) promotes HCC metastasis by competitively binding to miR-15a, miR-196a, or miR-196b and by subsequently increasing LAPTM4B (lysosomal-associated transmembrane protein 4B) expression (Figure 1D) [37]. LncRNA MALAT1 (metastasis-associated lung adenocarcinoma transcription 1) was reported to promote the migration and invasion of HCC by sponging miR-204 and releasing silent information regulator 1 (Sirt1) [38]. The high expression level of lncRNA HOXD-AS1 has been closely associated with a high tumor node metastasis stage in HCC patients. Mechanistically, lncRNA HOXD-AS1 competitively binds to miR-130a-3p, which can prevent SOX4 from miRNA-mediated degradation, thus activating the expression of EZH2 and MMP2 and can facilitate HCC metastasis [39]. In another study, intriguingly, lncRNA HOXD-AS1 up-regulated the Rho GTPase activating protein 11A (ARHGAP11A) via competitively interacting with miR-19a, leading to HCC metastasis [40]. In short, increasing studies show that the mechanism whereby lncRNAs act as ceRNAs controls the progression and metastasis of HCC.

Interestingly, some lncRNAs have been reported to promote or inhibit HCC metastasis by acting as molecular decoys to sequester miRNAs involved in EMT. A famous lncRNA ATB (activated by TGF-β), is a crucial regulator of the invasion–metastasis cascade, and can competitively bind with the miR-200 family and sequestrate the repression effect of the miR-200s on ZEB1/2, leading to EMT, cell invasion, and intravasation (Figure 1D) [41]. Some other lncRNAs, such as lncRNA HULC, linc-ROR, lncRNA-MUF, and MALAT1, have also been uncovered to act as miRNA sponges to regulate the expression of EMT markers in HCC. LncRNA HULC (highly upregulated in liver cancer) promotes the tumorigenesis and metastasis of HCC via enhancing the EMT progress in the miR-200a-3p/ZEB1 signaling pathway [16]. In a similar manner, linc-ROR induces EMT and promotes HCC metastasis via competitively binding miR-145, thus increasing ZEB2 levels [43] lncRNA-MUF (mesenchymal stem cell (MSC) upregulated factor), is one of the most significantly increased lncRNAs in HCC cells induced by HCC-MSCs. Mechanistically, it can indirectly drive EMT by competitively binding to miR-34a and upregulating SNAIL1 expression (Figure 1D) [42]. Similarly, lncRNA MALAT1 regulates the expression of ZEB1 by sponging miR-143-3p and promotes HCC progression [44]. These studies provide evidence that lncRNAs function as miRNA sponges, thereby suggesting that the lncRNA-miRNA-mRNA regulatory axis is pivotal for HCC metastasis.

#### 3.2.2. Interactions with mRNAs

Intriguingly, some lncRNAs also act on the processing of mRNAs to influence their stabilities and translation processes. LncRNA-ATB can stabilize and increase the mRNA of Interleukin-11 (IL-11) through the crosstalk with lncRNA-mRNA, thus promoting the colonization of disseminated HCC cells in distant organs (Figure 1E) [41]. In addition, PVTT (portal vein tumor thrombus) is a major complication encountered in HCC patients and it can be considered a special type of HCC metastasis [54]. A novel lncRNA ICR (ICAM-1 related) is up-regulated in PVTT tissues. Further analyses found that ICR enhances the ICAM-1 mRNA stability by forming an RNA duplex with it, thereby regulating the stem cell properties of ICAM-1+ HCC cells [45]. LncRNA MIR22HG (MIR22 host gene) repressed HCC metastasis by deriving miR-22 and binding with human antigen R (HuR) to increase MIR22HG stability, alter subcellular location of HuR, and decrease the binding abilities of HuR with oncogene mRNAs (Figure 1E) [28]. A liver-specific lncRNA LINC01093 suppresses HCC proliferation and metastasis by acting as a protein scaffold to recruit insulin-like growth factor 2 mRNA-binding protein 1 (IGF2BP1) to facilitate the degradation of GLI1 mRNA [29]. Collectively, these findings have indicated that lncRNAs may exert a wide variety of roles to impact HCC metastasis and progression by modulating the mRNA stability.

#### 3.2.3. Protein Modifications

Apart from the multiple aforementioned functions that allow for interactions of lncRNAs with miRNAs or mRNAs, lncRNAs also exert their biological functions by modifying proteins. Several studies have reported that lncRNAs may also be involved in the regulation of protein phosphorylation. A recent study revealed that lncRNA TSLNC8 (also known as LINC00589) exerts its tumor suppressive activity through the inactivation of the IL-6/STAT3 signaling pathway via physically interacting with TKT and STAT3, and thus inhibiting STAT3 phosphorylation and transcriptional activity in HCC [36]. Another study indicated that lncRNA HNF1A-AS1 (HNF1A antisense RNA 1) can inhibit the growth and metastasis of HCC by activating phosphatase through direct binding with the C-terminal of SHP-1 (SH2-containing protein tyrosine phosphatase-1) [30]. However, the effect of lncRNAs on gene expression through protein modification is not limited to the phosphorylation of target proteins. LINC01138 may exert its oncogenic activity through interacting with arginine methyltransferase 5 (PRMT5) and enhancing its protein stability by blocking ubiquitin/proteasome-dependent degradation in HCC (Figure 1F) [31]. LncRNA miR503HG, a host gene for the MIR503, specifically interacts with the heterogeneous nuclear ribonucleoprotein A2/B1(HNRNPA2B1), which suppresses metastatic tumor suppression through modulating the ubiquitination status of HNRNPA2B1 (Figure 1F) [32]. Another lncRNA uc.134, can suppress the migration and invasion of HCC by inhibiting the CUL4A (Cullin4A)-mediated ubiquitination and degradation of LATS1 (long-acting thyroid stimulator 1) in the cytoplasm [33]. Additionally, lncRNAs regulate protein degradation by influencing protein acetylation, an important post-translational protein modification. In another report, lncRNA-LET (low expression in tumor) is regulated by histone deacetylase 3 (HDAC3), which may be involved in hypoxia-induced cancer metastasis. Furthermore, lncRNA-LET reduced the degradation of the nuclear factor 90 (NF90) protein, which plays a pivotal role in hypoxia-induced cellular invasion [34]. Consequently, these studies indicate that lncRNAs may impact the development of HCC in diverse range of ways; interact with proteins to modulate post-translational modifications, such as phosphorylation or ubiquitination; and ultimately influence their activities and functions.

## 4. Emerging Paradigms on HCC Metastasis

### 4.1. Pathways Controlled by LncRNAs in HCC Metastasis

Currently, signal pathways affecting cell proliferation, invasion, and metastasis in HCC have been extensively studied. Furthermore, the involvement of lncRNAs in key carcinogenic or metastatic signaling pathways have been closely implicated [55].

Wnt/β-catenin signaling, which is the key mediator for the progression of malignancies [56], also plays a critical role in the development and metastasis of HCC cells [57,58]. Accumulating evidence suggests that the activation of the Wnt/β-catenin pathway could play a key role in HCC [59]. Actually, some lncRNAs have been experimentally demonstrated to be involved in the activation or inhibition of Wnt/β-catenin. For example, lncRNA-LALR1 enhances HCC cell cycle progression via recruiting CTCF (CCCTC-binding factor) to Axin1 promoter to block its transcription initiation and thus activates Wnt/β-catenin signaling [60]. LncRNA-MUF not only function as ceRNAs to regulate miRNAs, but also acts as a scaffold to enhance the interaction between GSK-3β (Glycogen synthase kinase-3β) and ANXA2 (Annexin A2), thus leading to the activation of Wnt/β-catenin signal transduction pathway and driving HCC metastasis [42]. Overexpressed linc00210 in liver cancer tissues can interact with CTNNBIP1 (catenin beta interacting protein 1), thereby blocking the inhibitory role of CTNNBIP1 in Wnt/β-catenin activation and promoting the interaction of β-catenin and the TCF/LEF complex, to activate Wnt/β-catenin signaling and liver tumor progression [61]. Moreover, lncFZD6 can also affect the Wnt/β-catenin signaling pathway, and drives Wnt/β-catenin activation through lncFZD6-BRG1-FZD6, thus promoting liver TIC self-renewal [21]. Lnc-FTX also inhibits HCC metastasis and invasion by upregulating the miR-374a target genes WIF1, PTEN, and WNT5A and repressing Wnt/β-catenin signaling activity [62].

The interactions of lncRNAs with other major signal pathways, such as STAT3, that are involved in the progression of HCC metastasis have been implicated [63,64]. For example, the lncRNA TSLNC8 and lncRNA-ATB mentioned above are related to the STAT3 pathway. LncRNA PTTG3P (pituitary tumor-transforming 3, pseudogene) promotes the growth and metastasis of HCC by up-regulating PTTG1 and activating PI3K/AKT signaling [65]. The lncRNA-AWPPH linc-GALH, which can promote the metastasis of HCC, is also involved in the regulation of AKT signaling pathway [18,35]. Furthermore, Linc00974 promotes the proliferation and metastasis via interacting with KRT19 (Keratin 19) in HCC. Further study shows that TGF-β signal pathways were substantially activated by the upregulation of KRT19 induced by Linc00974 [66]. Additionally, LncRNA uc.134 activates Hippo kinase signaling by inhibiting the translocation of CUL4A from the nucleus to the cytoplasm [33].

These studies highlight the diverse mechanisms by which lncRNAs can act as mediators of Wnt, JAK/STAT, PI3K/AKT, Hippo kinase, and other oncogenic signaling pathways. Nevertheless, how they affect the metastasis of HCC remains to be further elucidated, though the link between lncRNAs and signaling pathways has opened new window for the development of novel diagnostic and therapeutic applications in HCC.

### 4.2. Role of LncRNAs in the Multi-Step Process of HCC Metastasis

**Figure 2:**
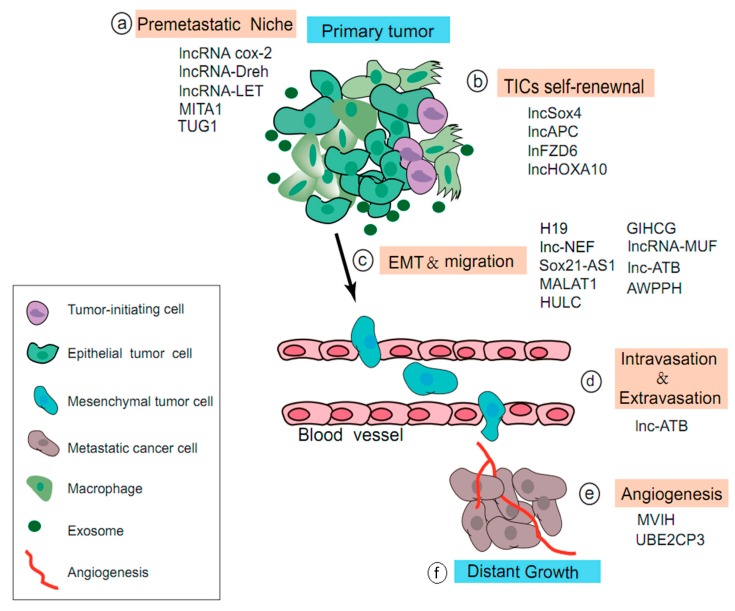
LncRNAs in the multi-step process of HCC metastasis. LncRNAs exert diverse regulatory roles in the multi-step metastasis of HCC, including (**a**) the pre-metastasis niche (e.g., lncRNA cox-2 and MITA1), (**b**) TIC self-renewal (e.g., lncSox4 and lncFZD6), (**c**) EMT and migration (e.g., H19 and Sox21-AS1), (**d**) intravasation and extravasation (e.g., lnc-ATB), (**e**) angiogenesis (e.g., H19 and Sox21-AS1), and (**f**) distant growth (e.g., MVIH and UBE2CP3).

Tumor metastasis is a complex and dynamic process involving the reciprocal interplay between tumor cells and host stroma from the microenvironment [67]. About 100 years ago, Stephen Paget described tumor metastasis by proposing the hypothesis of “seeds and soils” [68]. Nowadays, it is generally accepted that primary tumors may have already modulated the local microenvironment of distant organs in preparation for the colonization of tumor cells before their arrival. Presently, the microenvironment, which is termed the pre-metastatic niche, along with a series of changes—including inflammation, macrophage infiltration, hypoxia, and angiogenesis—may act in concert with lncRNAs and other various molecular events to orchestrate HCC metastasis. Thus, it has been recognized that lncRNAs may also be closely involved in the multistep metastasis of hepatocellular carcinoma. We summarize below the possible role of related lncRNA in terms of the pre-metastasis niche, cell differentiation, EMT, intravasation, extravasation, and angiogenesis (Figure 2).

In the pre-metastasis niche, lncRNA cox-2 prohibits immune evasion and metastasis of HCC by impeding the polarization of M1/M2 macrophages [69]. The hepatitis B virus X protein (HBx)-related lncRNA down-regulated expression by HBx (lncRNA-Dreh) attenuates HCC metastasis by targeting the intermediate filament protein vimentin [70]. In addition, lncRNAs also mediate tumor cells that exhibit unique metabolic phenotypes, such as lncRNA TUG1 (taurine up-regulated gene 1), which exerts a master regulator to coordinate glycolysis and metastasis in HCC [71]. Furthermore, emerging findings revealed that lncRNAs can modulate the transcription of metastasis-related genes to maintain TIC self-renewal and tumor initiation capacity, such as lncSox4 [23], lncFZD6 [21], and lncHOXA10 [22]. Moreover, lncRNA HULC and MALAT1 may promote HCC metastasis via enhancing EMT and migration in the miRNAs/ZEB1 signaling [16,38]. LncRNA-NEF and lncRNA-AWPPH can impede EMT progression and cancer metastasis via inhibiting the Wnt/β-catenin and PI3K/AKT pathway [26,35]. Another novel lncRNA MITA1, which is induced by energy stress, may promote EMT in a central step of HCC metastasis [72]. Intravasation and extravasation require the movement of cancer cells to the blood vessel, where lnc-ATB can regulate multiple steps of HCC metastasis, including EMT, invasion, and intravasation [41]. Notably, emerging studies show that the dysregulation of angiogenesis can be associated with HCC progression and metastasis. LncRNA UBE2CP3 [73] and MVIH [17] participate in HCC tumorigenicity and metastasis by modulating angiogenesis. MVIH could activate tumor-inducing angiogenesis by inhibiting the secretion of phosphoglycerate kinase 1 (PGK1); for example, MVIH expression was negatively correlated with the PGK1 level and positively correlated with the micro-vessel density in 65 cases of HCC.

Thus, summarizing the emerging link between lncRNAs and multi-step process of HCC metastasis will open up a new perspective on the role of lncRNA in modulating HCC metastasis, which may accelerate the progress of developing novel anti-metastasis strategies.

## 5. Potential Diagnostic and Therapeutic Applications

Although various diagnostic and treatment strategies are available for HCC, including surgical resection, liver transplantation, radio-frequency ablation, radiation therapy, radioembolization, and targeted therapies [74], most HCC patients are still usually diagnosed at the advanced stages, where there are limited treatment options and poor clinical effects. Metastasis is usually a major factor for the long-term survival of patients with advanced HCC. Therefore, it is urgent to explore new strategies for the early diagnosis, prognosis, and treatment of HCC. Several studies have shown that lncRNAs can be detected in the serum, blood, plasma, and urine of cancer patients [75,76]. Furthermore, lncRNA PCA3 has been approved by the FDA (Food and Drug Administration) as an early diagnostic biomarker of prostate cancer [77]. Many HCC-related clinicopathological parameters are found to be associated with lncRNAs, including the overall survival (OS) rate, PFS (Progression Free Survival), and metastasis rate. Notably, these characteristics enable lncRNAs to act as potential biomarkers for HCC. A study found that levels of lncRNA ZFAS1 are higher in HCC patients than in healthy controls, and in patients with cirrhosis and hepatitis B, and the expression of ZFAS1 is correlated with serum AFP (alpha fetoprotein). ZFAS1 could be identified as a novel serum biomarker for HCC diagnosis [78]. The researchers found that the up-regulation of MVIH could predict the frequent recurrence of early-stage HCC patients, suggesting that MVIH might be a potential biomarker for risk prediction and the individualized treatment screening of HCC patients after a hepatectomy. Moreover, it is also worth mentioning that recent studies have revealed that exosomal lncRNAs may also be important for predicting tumor invasion and metastasis, including HCC [79,80]. A previous study has provided strong evidence that the exosome-mediated transfer of lncRNA-TUC339 can modulate the adhesion of tumor cells and facilitate the migration of HCC [81]. Although there are only a few studies on circulating exosomal lncRNA biomarkers of HCC, tumor-derived exosomes contain tumor-specific lncRNAs, and their roles in cancer progression and metastasis are emerging. Combining lncRNAs and current biomarkers could be a feasible strategy to evaluate the efficacy and prognosis of HCC therapy.

Moreover, the possible role of lncRNA gene polymorphism in the risk of HCC was also explored, where data from a recent study demonstrated an inverse association of CASC8 gene polymorphisms, rs3843549, and rs13281615 with HCC progression and prognosis [82]. Collectively, lncRNAs gene polymorphism were associated with HCC with moderate epidemiological evidence and deserve further study and additional biological and clinical assessment.

Besides the imminent use of lncRNAs as biomarkers for diagnosis and prognosis, the therapeutic targeting of lncRNAs is also being explored. One of the approaches toward influencing lncRNA function is by utilizing specifically designed siRNAs (small interfering RNAs) against lncRNAs. Based on the mechanism of lncRNAs as molecular sponge of miRNAs. It is suggested that targeting diverse miRNAs using the artificial lncRNA could be a potential promising strategy for overcoming sorafenib resistance in HCC therapy [83]. Alternatively, antisense oligonucleotides (ASOs) have been proved to be able to regulate the coding genes link to a plethora of diseases, including solid tumors and lymphoma [84,85]. Preclinical studies have also demonstrated the therapeutic efficacy of ASOs targeting tumor-related lncRNAs [86]. We anticipate that the technological innovation of regulating lncRNA in vivo and in-depth study of lncRNA will help to develop a better therapy based on lncRNA to maximize the therapeutic potential. However, more clinical trials are needed to drive the development of lncRNA-based diagnostic tests and therapeutic interventions in order to benefit HCC patients.

## 6. Conclusions

The occurrence of HCC is a complex multi-gene and multi-step process. In the article above, we have highlighted the multifaceted regulatory mechanisms and signaling pathways of lncRNAs in HCC metastasis. LncRNAs execute a broad repertoire of functions involved in diverse molecule mechanisms, including epigenetic modification, transcriptional regulation, and post-transcriptional regulation. They are localized to specific cellular compartments, depending on their biological function. LncRNAs in the nucleus can function as versatile molecules via interacting with transcription factors or chromatin modifiers to regulate gene expression. Cytoplasmatic lncRNAs often act as regulators of post-transcriptional modulation, either acting as ceRNA or directly regulating mRNA stability. Additionally, we have summarized the current knowledge about the roles of lncRNA in regulating the tumor microenvironment and signal transduction pathways that can influence HCC metastasis, with the view of identifying novel strategies that may serve as future therapy for HCC.

Current treatment options for HCC, especially advanced HCC with metastasis, are extremely limited. Lack of highly specific and sensitive detection systems and appropriate therapeutic targets for HCC remain a major clinical challenge. As a participant in the metastasis of HCC, the new function of lncRNAs is still being explored, and it is expected to become a new potential target for cancer therapy and function as potent biomarkers to facilitate early diagnosis. Therefore, the emerging correlation between lncRNAs and HCC metastasis have opened up a more profound awareness of lncRNA-based diagnostics and targeted therapeutics.

## Figures and Tables

**Table 1:** Summary of long non-coding RNAs (lncRNAs) involved in the hepatocellular carcinoma (HCC) metastasis at different molecular levels.

LncRNA	Interaction Class	Interaction Partner	Expression of LncRNA	Pathway	Function	Mechanism	Reference
linc-GALH	RNA-TFs	DNMT1	Upregulated	AKT signaling	Promote metastasis	Epigenetically regulates Gankyrin by adjusting the ubiquitination status of DNMT1	[18]
lncRNA GIHCG	RNA-TFs	EZH2 DNMT1	Upregulated		Promote metastasis	Inhibits miR200b/a/429 transcript by recruiting DNMT1 and EZH2 to miR-200b/a/429 promoter	[19]
lncRNA SOX21-AS1	RNA-TFs	EZH2	Downregulated		Inhibit metastasis	Epigenetically silenced p21 via recruiting EZH2 to the promoter of p21	[20]
lncFZD6	RNA-TFs	FZD6	Upregulated	Wnt/β-catenin signaling	Promote metastasis	Interacts with FZD6 promoter and recruits BRG1 to initiate transcription	[21]
lncHOXA10	RNA-TFs	EZH2	Upregulated		Promote metastasis	Recruits SNF2L to the promoter to initiate the expression of HOXA10	[22]
lncSox4	RNA-TFs	Stat3	Upregulated		Promote metastasis	Drives Sox4 expression by recruiting Stat3 to be a Sox4 promoter	[23]
lncAPC	RNA-TFs	EZH2	Upregulated	Wnt/β-catenin signaling	Promote metastasis	Inhibits APC transcription by recruiting EZH2 to be a APC promoter	[24]
lncWDR26	RNA-TFs	SIX3	Downregulated		Inhibit metastasis	Inhibits WDR26 transcription by binding with SIX3	[25]
lncRNA-NEF	RNA-TFs	FOXA2	Upregulated		Promote metastasis	Interacts with β-catenin to increase the binding of GSK3β with β-catenin and inhibits phosphorylation of β-catenin	[26]
H19	RNA-protein	hnRNP U/PCAF/RNA Pol II	Downregulated		Inhibit metastasis	Associates with hnRNP U/PCAF/RNA Pol II and activates miR-200 family by increasing histone acetylation	[27]
MIR22HG	RNA-protein	HuR	Downregulated		Inhibit metastasis	Interacted with HuR to increase its stability	[28]
LINC01093	RNA-protein	IGF2BP1	Downregulated		Inhibit metastasis	Recruits IGF2BP1, preventing GLI1 binding to IGF2BP1	[29]
HNF1A-AS1	RNA-protein	SHP-1 C-terminal	Downregulated		Inhibit metastasis	Acts as phosphatase activator through interacting with SHP1	[30]
LINC01138	RNA-protein	PRMT5	Upregulated		Promote metastasis	Interacts with PRMT5 and enhances its protein stability	[31]
miR503HG	RNA-protein	HNRNPA2B1	Downregulated	NF-κB signaling	Inhibit metastasis	Interacts with the HNRNPA2B1 and modulates the ubiquitination status of HNRNPA2B1	[32]
lncRNA uc.134	RNA-protein	CUL4A	Downregulated	Hippo kinase signaling	Inhibit metastasis	Inhibits the translocation of CUL4A from the nucleus to the cytoplasm	[33]
lncRNA-LET	RNA-protein	NF90	Downregulated		Inhibit metastasis	Associates with NF90 to enhance the degradation of NF90	[34]
AWPPH	RNA-protein	YBX1	Upregulated		Promote metastasis	Promotes YBX1-mediated activation of SNAIL1 translation and PIK3CA transcription	[35]
TSLNC8	RNA-protein RNA-TFs	TKT STAT3	Downregulated	STAT signaling	Inhibit metastasis	Interacts with TKT and STAT3, and inhibits STAT3 phosphorylation and transcriptional activity	[36]
lncRNA HCAL	RNA-RNA	miR-15a miR-196a miR-196b	Upregulated		Promote metastasis	Binds to miR-15a, miR-196a, or miR-196b, and by increasing LAPTM4B expression	[37]
MALAT1	RNA-RNA	miR-204	Downregulated		Inhibit metastasis	Sponges miR-204 and release SIRT1.	[38]
HOXD-AS1	RNA-RNA	miR-130a-3p	Upregulated	MEK/ERK signaling	Promote metastasis	Binds to miR-130a-3p that prevented SOX4 degradation, activates the expression of EZH2 and MMP2	[39]
HOXD-AS1	RNA-RNA	miR19a	Upregulated		Promote metastasis	Upregulates the ARHGAP11A via bind to miR19a	[40]
lnc-ATB	RNA-RNA	miR-200 and IL-11 mRNA	Upregulated	TGF-β signaling	Promote metastasis	Binds with the miR-200 family and sequestrates the repression effect of the miR-200s on ZEB1/2; binds with IL-11 mRNA to promote organ colonization	[41]
lnc-MUF	RNA-RNA	miR-34a	Upregulated	Wnt/β-catenin signaling	Promote metastasis	Upregulate SNAIL1 expression	[42]
linc-ROR	RNA-RNA	miR-145	Upregulated		Promote metastasis	Sponges miR-145 to de-repress the expression of target gene ZEB2	[43]
MALAT1	RNA-RNA	miR-143-3p	Upregulated		Promote metastasis	Regulates the expression of ZEB1 by sponging miR-143-3p	[44]
lncRNA ICR	RNA-RNA	ICAM-1 mRNA	Upregulated		Promote metastasis	Regulates ICAM-1 expression by increasing the stability of ICAM-1 mRNA through RNA duplex formation	[45]

TFs: transcription factors; DNMT1: DNA methyltransferase 1; TICs: tumor-initiating cells; EZH2: Enhancer of Zeste Homolog 2; YBX1: Y-Box Binding Protein; FOXA2: Forkhead box A2; TKT: transketolase; STAT3: signal transducer and activator of transcription 3; HOXD-AS1: HOXD antisense growth-associated long noncoding RNA.
